# Comparison of Major and Minor Viral SNPs Identified through Single Template Sequencing and Pyrosequencing in Acute HIV-1 Infection

**DOI:** 10.1371/journal.pone.0135903

**Published:** 2015-08-28

**Authors:** Shyamala Iyer, Eleanor Casey, Heather Bouzek, Moon Kim, Wenjie Deng, Brendan B. Larsen, Hong Zhao, Roger E. Bumgarner, Morgane Rolland, James I. Mullins

**Affiliations:** 1 Department of Microbiology, University of Washington, Seattle, WA, 98195, United States of America; 2 Department of Medicine, University of Washington, Seattle, WA, 98195, United States of America; 3 Department of Laboratory Medicine, Seattle, WA, 98195, United States of America; 4 US Military HIV Research Program, WRAIR, Silver Spring, MD, 20910, United States of America; 5 Henry Jackson Foundation for the Advancement of Military Medicine, Inc., Bethesda, MD, 20817, United States of America; University of Athens, Medical School, GREECE

## Abstract

Massively parallel sequencing (MPS) technologies, such as 454-pyrosequencing, allow for the identification of variants in sequence populations at lower levels than consensus sequencing and most single-template Sanger sequencing experiments. We sought to determine if the greater depth of population sampling attainable using MPS technology would allow detection of minor variants in HIV founder virus populations very early in infection in instances where Sanger sequencing detects only a single variant. We compared single nucleotide polymorphisms (SNPs) during acute HIV-1 infection from 32 subjects using both single template Sanger and 454-pyrosequencing. Pyrosequences from a median of 2400 viral templates per subject and encompassing 40% of the HIV-1 genome, were compared to a median of five individually amplified near full-length viral genomes sequenced using Sanger technology. There was no difference in the consensus nucleotide sequences over the 3.6kb compared in 84% of the subjects infected with single founders and 33% of subjects infected with multiple founder variants: among the subjects with disagreements, mismatches were found in less than 1% of the sites evaluated (of a total of nearly 117,000 sites across all subjects). The majority of the SNPs observed only in pyrosequences were present at less than 2% of the subject’s viral sequence population. These results demonstrate the utility of the Sanger approach for study of early HIV infection and provide guidance regarding the design, utility and limitations of population sequencing from variable template sources, and emphasize parameters for improving the interpretation of massively parallel sequencing data to address important questions regarding target sequence evolution.

## Introduction

Sanger sequencing has been widely used to study the evolution of variable pathogens such as HIV, the emergence of drug resistance, and the rise of escape variants as a result of host immune pressures. Nonetheless, there are drawbacks associated with this technology. Individual template or cloned-derived sequencing is time-consuming, labor intensive, and is usually limited to tens of sequences per subject in consideration of cost. Consensus Sanger sequencing of virus populations can detect minority variants only above 10%- 25% of a heterogeneous sequence population [1] and with five individual Sanger sequences, the probability of observing a variant that represents at least 10% of the viral population is only 40% [2]. This resolution threshold is restrictive, especially when investigating minor HIV-1 variants. Massively parallel sequencing (MPS) technologies such as pyrosequencing, which involve individually amplifying and sequencing large numbers of DNA template molecules, have been applied extensively in HIV-1 research in an attempt to identify the presence of minority variants, particularly those relating to the emergence of clinically relevant HIV-1 drug resistance [3–7] and immune escape variants [8–13].

Four factors determine the level of minor variant resolution in MPS technologies such as 454-pyrosequencing, the related Ion Torrent system, and the Illumina platforms: a) error rate associated with the initial PCR amplification rounds (and cDNA synthesis, in the case of RNA templates) prior to sequencing; b) accurate quantitation of the number of amplifiable input template molecules; c) number of pyrosequences (reads) that map to the genomic position with the observed polymorphism; and d) resolution of errors that are inherent to the sequencing process.

Incorrect base incorporation by DNA polymerases during cDNA sysnthesis and PCR amplification has the potential to introduce errors within DNA products prior to pyrosequencing thus making these mismatch errors indistinguishable from real variation after sequencing. These PCR introduced errors have been shown to introduce biases when characterizing microbial community structure [14–16] and have an impact on detection of rare HIV-1 sequence variants [17, 18]. We have shown previously that current pyrosequencing error correction algorithms perform with reduced specificity in identifying PCR introduced mismatch errors [19]. Thus, when looking for rare genetic variants, it is critical to use an enzyme with high fidelity and optimized conditions to ensure that the genetic variation found within the sequence population is representative of the virus and not an artifact of the amplification process [20].

While the term “coverage” is most often used to refer to the number of reads mapped to a genomic position, this quantity alone cannot be used to gauge the number of actual viral templates sequenced using MPS technologies. Indeed, careful estimation of the number of amplifiable templates is rarely performed, but is essential to accurately measure population diversity [21–24]. The metric “sequencing depth”, defined as the number of reads mapped to a genomic position divided by the number of estimated genome templates in the sequencing reaction, was used in this study to represent template coverage. The read coverage obtained through library sequencing is often uneven [20, 25], as is amplicon sequencing near the ends of the amplicons [20], hence, sequencing depth can vary greatly by nucleotide site across a sequenced region.

Errors within pyrosequences also follow distinct patterns compared to traditional Sanger sequencing. Consecutive runs of the same nucleotide (homopolymers) are particularly error-prone, resulting in inclusion of more or less bases in the read than is actually present in the DNA template. In pyrosequences generated by the GS-FLX Titanium technology, the mean homopolymer-associated error has been estimated at 1.1%, with errors showing a non-random distribution and certain positions showing error rates as high as 50% [26]. Sanger sequencing has an estimated per-base error rate of <0.1% [27, 28]. The relatively higher error rate in pyrosequences further complicates distinguishing real variants from sequencing artifacts [29–32].

Over the past few years, several error correction algorithms for identifying and correcting pyrosequencing artifacts have been described [19, 33–42]. Despite the availability of these algorithms to identify minor HIV-1 variants, one should proceed with caution when asserting their biological significance as they approach the level of error rates.

There have been studies comparing the prevalence of low frequency (<20%) HIV-1 drug resistance mutations within clinical samples sequenced through Sanger sequencing, especially when determining consensus bases at each position, and pyrosequencing [43–47]. These studies highlight the concordance between the two sequencing technologies when comparing higher frequency drug resistance mutations, but also discuss the presence of lower frequency (<20%) clinically relevant drug resistance mutations within pyrosequences. HIV-1 genetic diversity has also been compared with Sanger and pyrosequencing [48]. While these studies report minor variants observed uniquely within pyrosequences, most but not all [24] do not report details regarding the amplifiable viral templates prior to pyrosequencing, which further obfuscates estimation of minor variant frequencies. Additionally, authors reporting minor HIV-1 variants at a frequency range of 0.1%- 5% in pyrosequences often do not factor in the actual number of viral templates sequenced [11, 13, 34], and thus have not estimated the true population frequency of those variants. Indeed, some studies have sought to have the number of templates in excess of the number of sequence reads [49, 50], a protocol that further obscures the validity of individual sequences. One study that compared major and minor HIV-1 SNPs in a population of chronically HIV- infected individuals reported multiple instances of major HIV-1 variants (found in ≥50% of sequences) from pyrosequences that were not observed in Sanger sequences [48]. The probability of this, even with a limited number of Sanger sequences (e.g., five), is less than 20% [2]. However, as no quantitation of templates was done in that study, it is unclear whether pyrosequencing was performed on the same number of templates as were used in Sanger, or many times more, which could explain the discrepancy in the variants observed.

In the present study we comprehensively compared the minor and major SNP variations observed in Sanger sequences and pyrosequences across three HIV-1 genomic regions, *gag*, *gp120* and *nef*, in 32 subjects who became infected with HIV-1 during the MrkAd5 Step vaccine trial [51]. We performed this comparison with the goal of determining whether greater depth of population sampling afforded by pyrosequencing allowed detection of additional minor variants in instances when Sanger sequencing detects only a single variant founding the infection. We assessed the concordance between SNP frequencies in both sequencing technologies, and the effect of pyrosequencing error-correction algorithms on minor variant frequencies. We also investigated whether minor SNP variants specifically observed in pyrosequences were more frequently adjacent to error-prone regions, namely homopolymers [18, 26]. Finally, we assessed the impact of sequencing depth and the number of Sanger sequences on the concordance and resolution of minor variants

## Materials and Methods

### Study subjects

All 32 subjects were in early HIV-1 infection (within 1.5 months of the first HIV-positive visit) and enrolled in the MrkAd5 Step HIV-1 vaccine trial (Clinical Trial Identifier: NCT00095576), a double-blind phase IIb test-of-concept study of the Merck Adenovirus-5 (MRK Ad5) HIV-1 clade B vaccine with *gag*, *pol* and *nef* inserts [51–53]. Institutional human subjects review committees at each of the clinical sites approved the vaccine protocol prior to trial initiation, and all study participants provided written, informed consent. At specified collection dates during the trial, PBMC and plasma samples were collected from the enrolled subjects. PBMC samples from the subjects included in this study were collected following vaccination but prior to HIV-1 infection. The first available HIV-1 positive plasma samples from were sequenced by two sequencing methods: individual template sequencing via Sanger sequencing and pyrosequencing. The subjects were part of a larger group of infected trial subjects and the sequencing of the first available HIV-1 positive plasma samples is part of an ongoing study to characterize genetic signatures of vaccine-induced immune pressure on breakthrough HIV-1 sequences (manuscript in preparation). The trial subjects examined in this study included 13 placebo and 19 vaccine recipients.

### Sanger sequence polymorphism analysis

The Sanger sequences used in this study were derived from single amplifiable near-full-length viral genome (NFLG) and half genome templates, and have been deposited in GenBank under accession numbers JF320002-JF320643 [51]. Sequences were quality-checked and used to generate a multiple-sequence alignment using the HIV-1 strain HXB2 as the reference sequence. A consensus sequence was then generated for each subject and used as reference to realign the sequences. The web tool InSites (http://indra.mullins.microbiol.washington.edu/DIVEIN/insites.html) [54] was used to identify the positions of SNPs in the aligned sequences. For the comparison to pyrosequences, InSites was used to distinguish positions with SNPs present in a single Sanger sequence (private sites) and those shared by more than one sequence (phylogenetically informative sites). Details about the number of templates sequenced per subject are given in S3–S5 Tables.

### Identification of founder variants

The number of variants establishing productive infection (henceforth referred as founders) for each subject was identified from Sanger sequences based on phylogenetic and genetic distance analyses [51]. Probable multiple founders were identified based on shared polymorphisms (ranging in this set between 1–4), occurring in groups of at least two sequences, that were not shared with the remaining sequences [51]. A total of six of the 32 subjects were identified as having been infected with multiple founders.

### Nucleic acid extraction for pyrosequencing

Using the plasma samples from the same visit date as the previously derived Sanger sequences, RNA was extracted using the Qiagen Viral RNA Mini Kit (Qiagen, Valencia, CA). cDNA was synthesized using Superscript III Reverse Transcriptase (Invitrogen, Grand Island, NY), over three 1.5kb regions corresponding to *gag*, *gp120*, and *gp41-nef*, using the first-round reverse PCR primer. The list of primers used is provided in S1 Table. The three gene regions were selected for pyrosequencing: *gag*, *env* and *nef*. The MrkAd5 vaccine insert included *gag* and *nef* and hence these gene regions were selected for studying impact of vaccine-induced immune responses. The *env* region was not part of the vaccine, and this was selected as a control.

### PCR amplification

PCR amplification prior to pyrosequencing was done using Advantage LA or Advantage 2 DNA Polymerase (Clontech, Mountain View, CA). The viral template input was estimated using clinical viral load measures. First round PCR was a multiplex reaction, using primers to simultaneously amplify all three non-overlapping genomic regions, *gag*, *gp120*, and *gp41-nef*. The second round of PCR was done separately for each gene using nested primers (S1 Table). Endpoint dilution was performed to approximate the number of amplifiable viral copies per gene using the Quality template-estimating program [22] (http://indra.mullins.microbiol.washington.edu/quality/). Once amplifiable template numbers were determined, additional PCR reactions were performed to amplify a target of up to 5000 templates for specimen. PCR reactions were subsequently cleaned using Agencourt AMPure XP beads (Beckman Coulter, Brea, CA) and DNA concentrations were determined spectrophotometrically. Products from all three gene regions from individual study participants were pooled for 454-pyrosequencing.

### Library preparation and pyrosequencing

Pooled and purified PCR amplified products were quantified using the Quan-it PicoGreen dsDNA assay (Invitrogen). GS-FLX Titanium kits were used for Rapid Library Preparation and Rapid Library MID Adaptor addition (Roche, Branford, CT). 500ng of each sample was nebulized, end repaired, and ligated with 454 library adaptors and MIDs. Fragments between 600–900bp were selected for and purified using AMPure beads. Library quality was assessed using the Agilent High Sensitivity DNA Bioanalyzer kit and chip (Santa Clara, CA), and the quantity of DNA was measured using the Quan-It PicoGreen dsDNA assay. Library concentrations were calculated using the online Roche Rapid Library Quantitation calculator. Each DNA library was diluted to a working stock of 1x10^7^ molecules/μl in TE buffer. Libraries generated from multiple samples (each with distinct sequence tags) were mixed at equimolar ratios. Emulsion PCR (Roche) was performed on the combined libraries using a ratio of 2–3 DNA molecules per bead. PCR-positive beads (~10–20% of emulsion PCR products) were then selectively enriched. Four million enriched beads were loaded onto a 454 picotiter plate and pyrosequences were generated using the 454 GS FLX system. Median number of pyrosequences generated per gene region for the subjects in this study is given in S3–S5 Tables.

### Pyrosequence data cleaning

Pyrosequences and their associated signal intensities were processed using the error correction program CorQ [19]. Briefly, signal intensities were clustered and corrected with AmpliconNoise [33, 37] for an initial improvement of insertion and deletion (indel) and SNP errors. A reference-based multiple-sequence alignment with the corrected sequences was generated for each gene using the subject consensus from the Sanger sequences [51]. Reduced read coverage and sequence quality after pyrosequencing, as well as sample availability limitations, led to the *gp41* region region being excluded in subsequent variant analyses. Following the construction of multiple-sequence alignments, a collection of Perl programs [19] were run on the aligned sequences and associated base-quality files to identify and correct regions with poor quality in a sequence-context dependent manner. Indel errors that resulted in frameshifts were corrected. Additionally, SNPs observed in only a single read were corrected to match the consensus at that position. No further mismatch error correction was applied. A detailed description of the error correction algorithm and parameter settings is provided in Supplementary materials.

### Mismatch frequency threshold

The sensitivity of minor variant detection in pyrosequencing experiments is determined in part by the PCR conditions used to generate the templates for pyrosequencing, and the number of amplifiable templates in the reaction. Subsequent to the PCR amplifications done for this study, a number of DNA polymerases and varying PCR conditions were assessed to identify differences in sensitivity and mismatch error-rates [20]. Based on initial sensitivity estimates, the DNA polymerase enzymes used in this project, Advantage LA and Advantage 2 (Clontech), were found to have high sensitivity, but mismatch error-rates as high as 1% [20]. As currently available pyrosequencing error-correction programs are not equipped to filter out mismatch errors generated during PCR amplification [19], a frequency threshold of 1% was used as the limit of detection for all SNP analyses.

### Terminology

Minor SNP variants were those observed at a frequency between 1–50% in the sequences of a given subject. A major variant difference was defined as a polymorphism at a position in which the consensus base varied between the two sequencing methods. In most cases, the differences in major variants were due to a “frequency reversal” of the two relatively abundant variants. SNPs observed only in pyrosequences or Sanger sequences were classified as pyrosequencing-specific (PS-SNPs) or Sanger-specific (SS-SNPs) SNPs, respectively. Shared minor SNPs are nucleotide differences from the consensus that occur at the same genomic location in both the Sanger and pyrosequences in a given study subject.

### Sequencing depth

The metric sequencing depth is defined as the number of reads mapped to a genomic position (read coverage) divided by the number of estimated amplifiable genomic templates in the sequencing reaction. PCR amplification following end point dilution of templates is performed to estimate the number of amplifiable viral copies per gene using the quality template-estimating program [22] (http://indra.mullins.microbiol.washington.edu/quality/). Sequencing depth is used as a measure of template coverage. The read coverage is the number of reads that map to a genomic position and is determined after a reference based multiple sequence alignment of pyrosequencing reads. Mean number of amplifiable templates and mean sequencing depth for each subject is given in S3–S5 Tables.

### Statistical methods

Spearman’s rank correlation coefficient (ρ) was used to estimate correlation between SNP frequencies. Kruskal-Wallis tests were performed to compare the correlation among multiple groups with Dunn’s error correction for multiple comparisons. The non-parametric Mann-Whitney test was used to compare two distributions.

## Results

**Fig 1:**
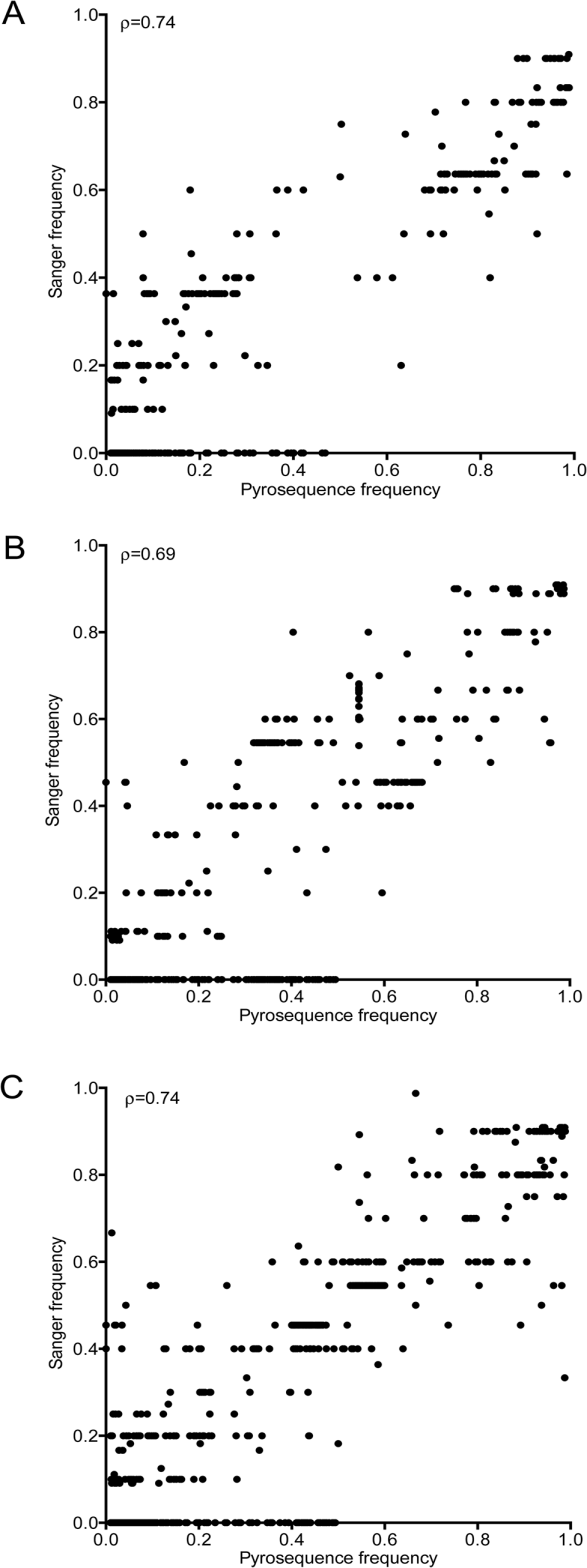
Correlation between SNPs observed in Sanger and pyrosequencing datasets. SNP frequencies are shown for *gag* (A), *gp120* (B), and *nef* (C). All types of SNPs evaluated (shared, Sanger-specific, and pyrosequencing-specific) from all 32 subjects are shown. Spearman’s correlation coefficients are noted for each comparison.

**Fig 2:**
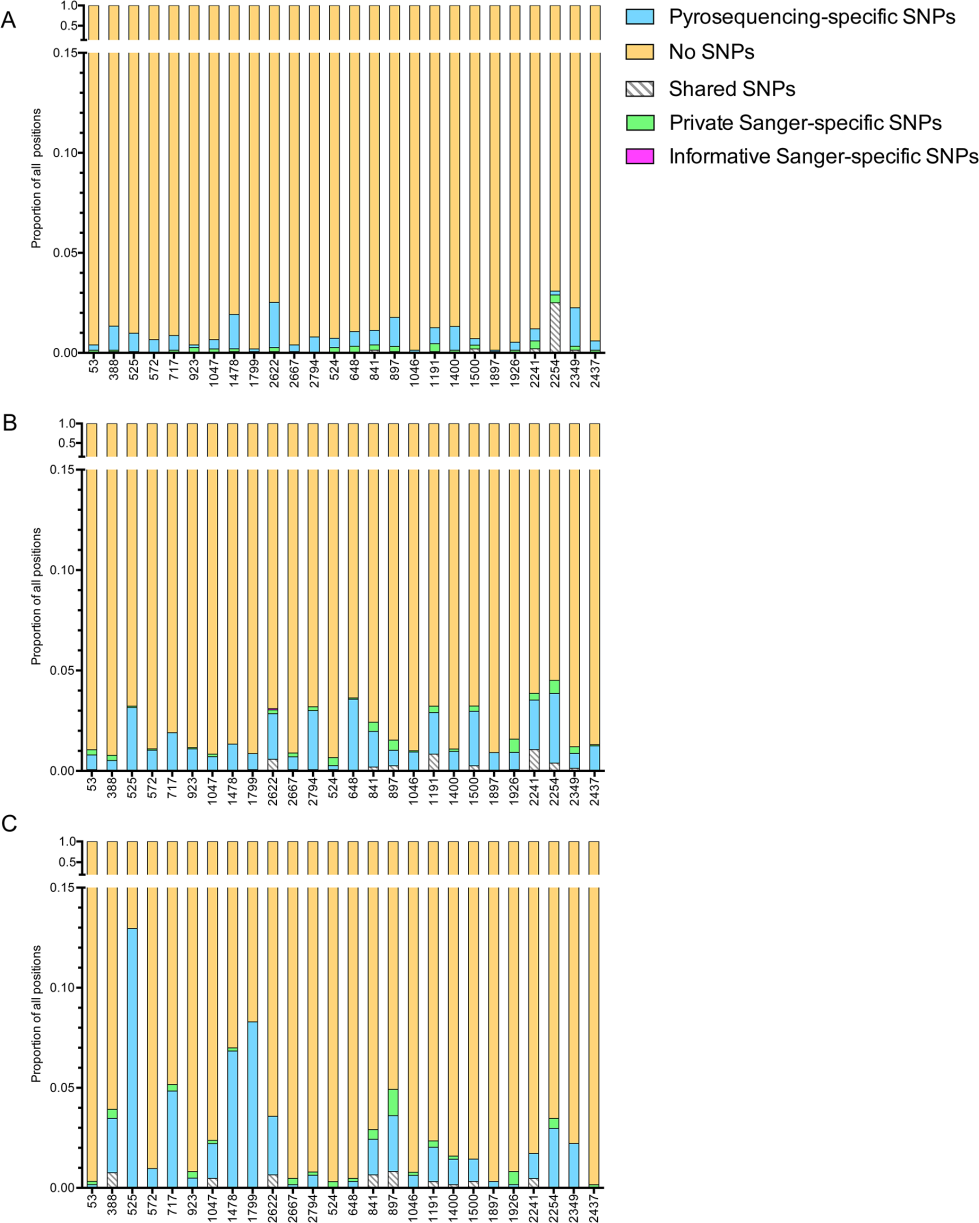
Proportion of positions with and without SNPs in subjects with a single founder virus. The Y-axis shows the proportion of nucleotide positions in *gag* (A; 1500nt), *gp120* (B; 1530nt), and *nef* (C; 615nt) that correspond to each category, with a linear scale and a split at 0.15. The X-axis indicated each subject ID (502-XXXX). The key shows the type of SNP observed. Data for subjects with multiple founders is shown in S1 Fig.

Overall, a strong correlation in SNP frequencies was found between Sanger and pyrosequencing data sets (Fig 1). Fig 2 illustrates SNP frequencies in the 26 subjects with a single founder variant. As expected for subjects within 1.5 months of HIV-1 acquisition, the majority of the positions (>97%) along the three genes had no observable polymorphisms. Subjects designated as having multiple founders had fewer non-polymorphic sites (92–97%), S1 Fig Only one subject with a single founder (502–2622) had a phylogenetically informative (found in more than one sequence) SS-SNP detected (in *gp120*). As expected, private (found only in one sequence) SS-SNPs and PS-SNPs were more prevalent across all gene regions in the individuals with replicating multiple founders (compare Fig 2 to S1 Fig).

### Major variant comparisons

A consensus nucleotide sequence was generated from both pyrosequences and Sanger sequences for each subject over the 1500, 1530, and 610 nucleotide regions in *gag*, *gp120*, and *nef*, respectively. Among subjects infected with a single founder, the pyrosequence- and Sanger-derived consensus sequences were identical for 21 of the 26 subjects in *gag* (80%), 23 in *gp120* (88%), and 22 in *nef* (84%), with the number of positions with consensus mismatches ranging from 1–3 (median = 1), S2 Fig. In subjects with one or more consensus mismatches, there was an overall nucleotide identity of >99% in the consensus sequences. There were only two subjects (7%) in which a consensus base from pyrosequencing was absent in the five and six Sanger sequences available for these subjects, respectively. All other instances of consensus mismatches were due to frequency reversals between shared major and relatively abundant minor variants. There were no cases of the consensus Sanger variant being absent from the pyrosequencing dataset, and we found no evidence of consensus base discrepancies resulting from incomplete indel error correction of pyrosequences.

Not surprisingly, for subjects with multiple founders, the consensus sequence concordance was lower, with all consensus base mismatches resulting from frequency reversals between the two most common variants (S2 and S3 Figs). In addition, no relationship was found between the frequency of a variant in the pyrosequences and primer sequence homology to that variant (data not shown).

### Minor SNP variant comparison

**Fig 3:**
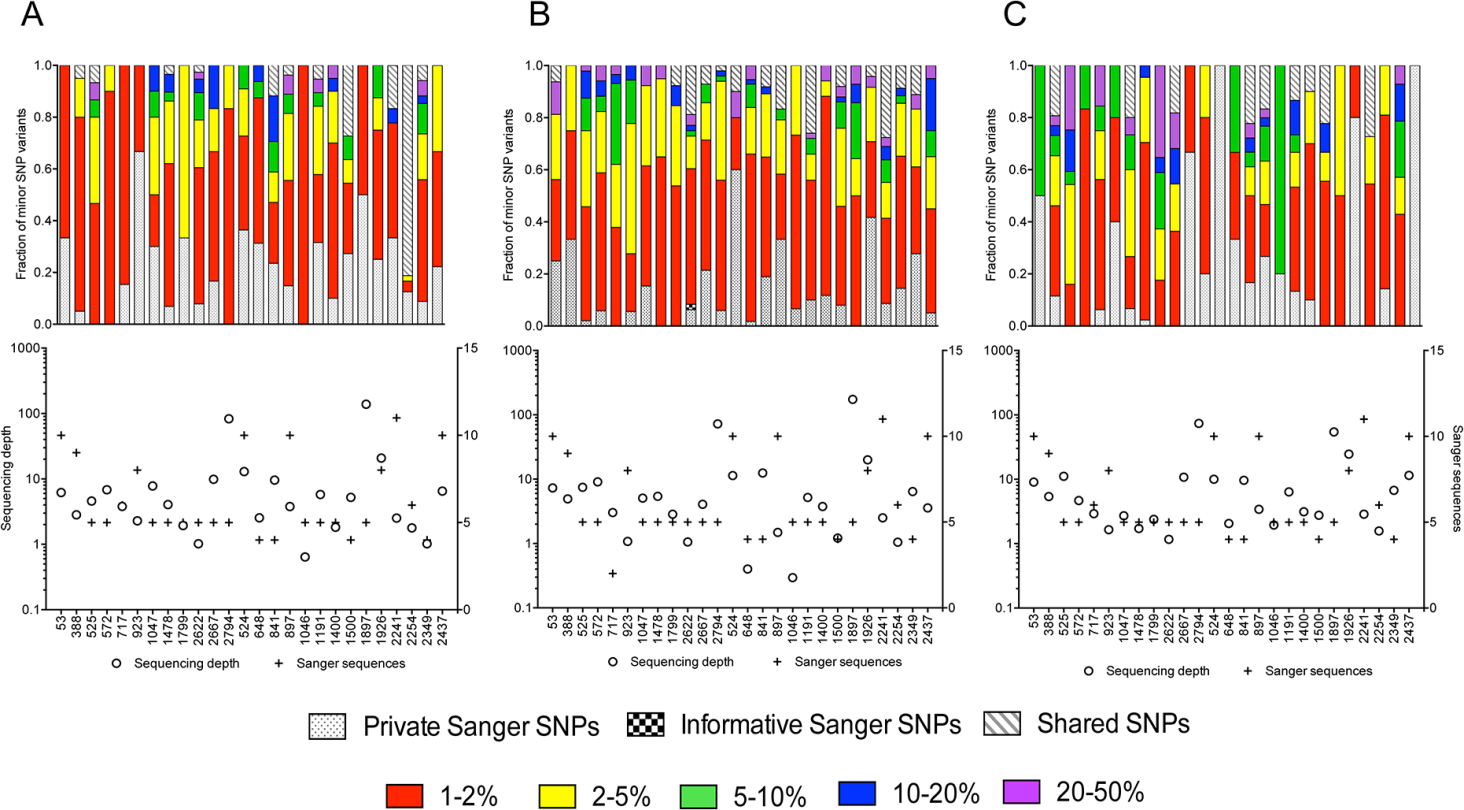
Frequencies of minor SNPs. Minor SNPs (frequencies between 1–50%) were compared in the 26 subjects with a single founder virus across *gag* (A), *gp120* (B) and *nef* (C). The upper panels indicate for each subject the proportion of minor SNP variants in each category. The categories included shared (found in both Sanger and pyrosequences), Sanger-specific, including those that were Private (found in 1 sequence) and Informative (found in 2+ sequences), or found only in pyrosequences (with frequencies indicated by color: 1–2%, red; 2–5%, yellow; 5–10%, green; 10–20%, blue; 20–50%, purple). The lower panels show the pyrosequencing depth (o, left y-axis), defined as number of reads mapped to a position divided by mean number of amplifiable viral templates, and the number of Sanger sequences (+, right y-axis). The X-axis lists the subject publication ID (502-XXXX)[51]. Comparable data for subjects replicating multiple founder viruses is shown in S4 Fig.

Fig 3A–3C shows the frequency distribution of minor SNP variants in subjects with single founders. Most minor PS-SNPs (63%, 56%, 42% in *gag*, *gp120* and *nef*, respectively) represented <2% of the sequence population in subjects with single founders (Table 1). Similar distributions were found in subjects with multiple founders, although as expected, a higher fraction made up between 20–50% of the sequence population (S4A–S4C Fig).

**Table 1:** Frequency of minor pyrosequence-specific SNP variants. Shown is the frequency distribution of minor PS-SNPs (between 1–50%) as a fraction of the minor SNPs observed within that gene across all subjects, separated into those replicating single versus multiple founder viruses early in infection.

	Pyrosequencing-specific minor SNP frequencies				
**Single Founder**	**1–2%**	**2–5%**	**5–10%**	**10–20%**	**20–50%**
*Gag*	63.23	21.65	7.22	4.81	3.09
*gp120*	56.60	27.72	7.92	3.96	3.80
*Nef*	42.25	25.13	10.43	7.75	14.44
**Multiple Founder**	**1–2%**	**2–5%**	**5–10%**	**10–20%**	**20–50%**
*Gag*	46.51	18.60	9.30	13.95	11.63
*gp120*	42.28	17.45	12.08	7.38	20.81
*Nef*	20.65	8.70	3.26	0.00	67.39

To ensure that the observed PS-SNPs were not the result of artifactual mismatch errors adjacent to homopolymer regions [26, 42], the sequence context of SNPs were assessed and no difference was found in the distribution of mismatches between homopolymer and non-homopolymer regions (p = 0.23, S5 Fig). Minor variant resolution within pyrosequences also depends on correctly estimating the number of amplifiable templates, as well as the number of reads mapping to each genomic position [20]. When we considered only positions at which the sequencing depth was at least one (the number of reads was equal to, or greater than, the number of amplifiable templates used to derive products for the sequencing reaction), the number of positions with minor PS-SNPs was reduced by an average of 51% (S6A–S6C Fig). We also quantified the number of PS-SNPs observed at a frequency below the expected Sanger sequencing threshold across all subjects and found that on average 89% of all PS-SNPs in all three gene regions were present below the detection threshold for Sanger sequencing (S2 Table). Of those, 81% in *gag*, 80% in *gp120*, and 60% in *nef* were present in <5% of pyrosequences.

Within pyrosequences, we found phylogenetically-informative Sanger SNPs well-represented, with only one informative SNP missing within the pyrosequences. In this particular case there were an inadequate number of reads covering that site (sequencing depth <0.1). Among those infected with multiple founders, only one subject had informative SNPs that were absent in the subject’s pyrosequences (S4 Fig). However, only one of 42 positions had a sequencing depth of <1 (S7 Fig) and thus, their absence from the pyrosequencing data was not due to low coverage. As estimated previously for these subjects [51] a median of 2, 3 and 1 private Sanger SNPs were present in *gag*, *gp120* and *nef*: of these, 70% were observed below the 1% threshold within the subject pyrosequences (S8 Fig), while 30% of these private Sanger SNPs were not found within the subject pyrosequences at all.

To understand the effect of sequencing depth on resolution of minor frequency sequencing artifacts, we investigated the correlation between frequencies of PS-SNPs observed in positions with sequencing depths ranging from <1 to >10 and observed no significant association between the mean PS-SNP frequencies observed in positions with varying sequencing depths in *gag* and *nef*. In *gp120* there was a significant (p < 0.05) increase in mean PS-SNPs frequencies when comparing positions with depth of < 1 to positions with depth > 5 (S12 Fig).

### Shared SNPs distribution and frequency concordance

**Fig 4:**
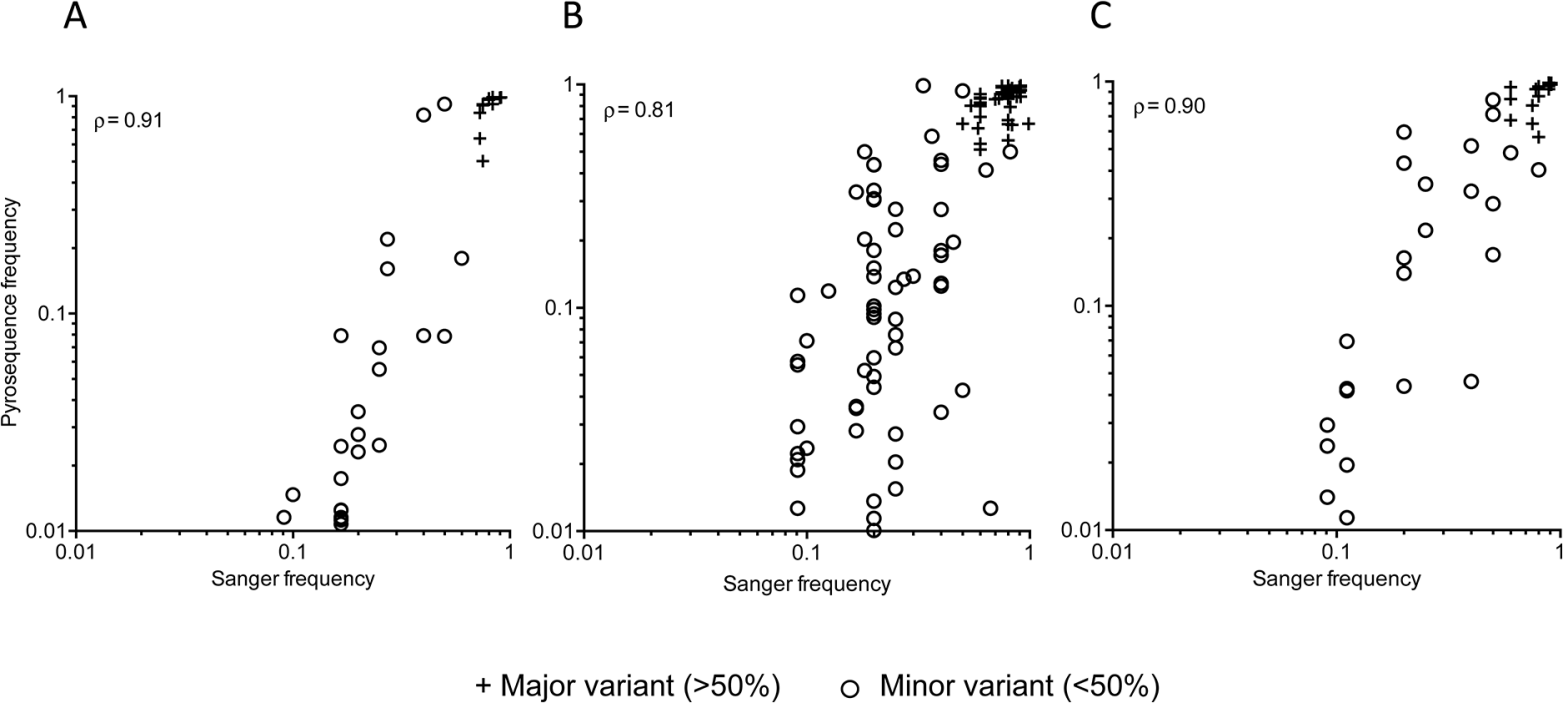
Comparison of frequencies of SNPs shared between Sanger and pyrosequences. (A) *gag* (n = 54 SNPs), (B) *gp120* (n = 64), and (C) *nef* (n = 24). Major (+) and minor (o) variant frequencies are plotted for subjects with a single founder, including positions at which the major and minor SNP frequencies were reversed in the two sequencing sets. Spearman correlation coefficients are shown for each comparison. The same analysis for subjects replicating multiple founders is shown in S9 Fig.

Individuals with multiple founders, as expected, had a higher fraction of positions with shared SNPs found in both sequencing platforms (*gag*: p = 0.0003, *gp120*: p = 0.0009, *nef*: p<0.0001). There was also high concordance between the frequencies of both shared major and minor variants in individuals with single (Spearman’s ρ = 0.91 for *gag*, ρ = 0.81 for *gp120*, ρ = 0.90 for *nef*, p<0.0001 for all three regions, Fig 4) and multiple founders (ρ = 0.93 for *gag*, ρ = 0.91 for *gp120* and ρ = 0.44 for *nef*, p<0.0001 for all three regions, S9 Fig).

**Fig 5:**
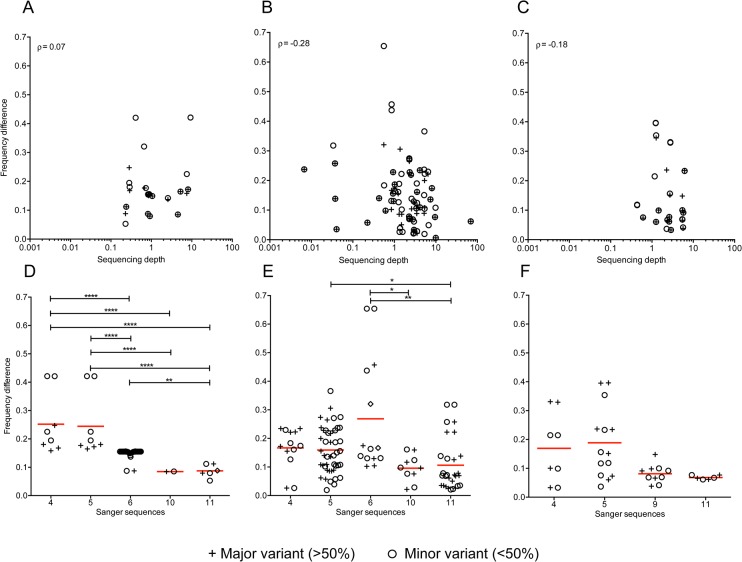
Effect of sequencing depth on frequency correlations between shared minor SNPs in individuals with single founder variants. (A-C) Associations between the absolute frequency difference and the pyrosequencing depth of the corresponding position in the 54, 64, and 24 shared SNPs found in *gag* (A), *gp120* (B) and *nef* (C) sequences. Spearman correlation coefficients are shown for each comparison. (D-F) Correlations between the number of Sanger sequences and the absolute frequency difference for shared SNPs. Bars above each panel indicate the significance of the correlation (* <0.05. ** <0.01, *** <0.001, **** <0.0001) using a Kruskal-Wallis test with Dunn correction for multiple comparisons. Data for subjects with multiple replicating founder variants is shown in S10 Fig.

Higher sequencing depth would be expected to afford better agreement in variant frequencies between the two sequencing datasets. Among subjects with single founders, we observed no significant trend between frequency concordance and sequencing depth in *gag*, and *nef* (ρ = 0.07, p = 0.49 and ρ = -0.18, p = 0.23 respectively). In subject *gp120* sequences, we observed reduced frequency difference with increasing sequencing depth (ρ = -0.28, p = 0.002) (Fig 5A–5C). In subjects with multiple founder variants, no correlation between frequency concordance and sequencing depth were observed in *gp120* and *nef* (ρ = 0.08, p = 0.07 and ρ = 0.21, p = 0.0006 respectively, S10). In *gag* sequences we observed reduced frequency differences with increasing sequencing depth (ρ = -0.32; p = 0.0008, S10 Fig). When sequencing depth was applied as a filter for shared SNPs below a depth of 1, the number shared SNPs was reduced by 12–19% (S11 Fig).

Increasing the number of Sanger sequences from five should improve minor variant frequency resolution. Fig 5D–5F shows the number of Sanger sequences generated for a subject with the corresponding absolute difference in shared SNP frequency, for both consensus and minor variants. In subjects with a single founder variant, a significantly increased frequency concordance was observed between those with ≥10 Sanger sequences compared to subjects with ≤5 Sanger sequences in *gag* (p<0.0001, Fig 5D) and for those with ≤6 vs. ≥10 Sanger sequences in *gp120* (p<0.05, Fig 5E). A similar but non-significant trend was observed in *nef*. Among subjects with multiple founders, the results were similar, except that in *nef* there was a trend towards less concordance with more Sanger sequences (≤5 vs. 11, p = 0.01). However, a single subject with 11 Sanger sequences largely drove this result as this subject had a large proportion of positions with frequency reversals (S3 and S10 Figs).

Finally, we compared SNP variation between subjects in vaccine and placebo groups to look for any differences based on treatment assignment. We compared the fraction of SNPs shared between the two sequencing methods, ones observed specifically within pyrosequences and ones observed specifically within Sanger sequences across subjects in the vaccine and placebo groups and found no statistically significant differences the two groups (S6 Table).

## Discussion

Single-nucleotide polymorphisms observed in pyrosequencing data were compared to those observed in Sanger sequences in order to determine the concordance between the two technologies, and to assess the quality and utility of the information provided by the greater depth of massively parallel sequencing when assessing HIV founder populations, i.e., those viruses establishing infection. The majority of subjects infected with single founder variants had no differences between consensus bases from the two sequencing sets. In subjects with consensus disagreements, less than 1% of the sites surveyed had discordant bases between the two sequencing sets. Consensus differences were generally associated with decreased numbers of Sanger sequences or frequency reversals between major and frequently observed minor variants. There was no evidence that primer mismatches led to preferential amplification of one variant over another in subjects with multiple founders that would explain the rare consensus base differences observed.

As a component of the strength of massively parallel sequencing technologies lies in the detection of minor variants, the distribution of minor SNPs was assessed—a majority (>53%) of the PS-SNPs observed were rare in the viral sequence population (<2%), whereas only 9% of the PS-SNPs observed in pyrosequences were found in more than 25% of the sequences. That the majority of SNPs fell below 2% of sequence population is not surprising given the large number of reads generated through pyrosequencing. Based on above results from consensus and minor variant comparisons we found that founder sequences identified from 5 single template-derived sequences did not differ from those established through pyrosequencing. Furthermore, the greater depth afforded by MPS technology did not results in the detection of additional minor founder variants in the subjects where Sanger sequencing identified only a single founder establishing infection.

Only one informative Sanger SNP (from a subject infected with a single founder, and with the SNP found in 2 out of 5 sequences) was absent in pyrosequences. However, the pyrosequencing depth at this position was less than 0.1, which likely explains its absence. Among subjects with multiple founders, only one subject had phylogenetically informative SS-SNPs, despite the majority of positions having a pyrosequencing depth of at least one. This subject also showed the highest number of consensus base mismatches of all subjects. These differences could be the result of using different plasma vials and cDNA preparations, or an indication of the stochastic nature of quantitation of multiple viral variants. All other phylogenetically informative Sanger SNPs were observed in the pyrosequencing dataset above the 1% threshold. This result lends confidence that all the consensus viral templates observed within the Sanger sequence population were also adequately sequenced by pyrosequencing. Private-site Sanger SNPs were found at a frequency of 0.22% per nucleotide sequenced. Interestingly, 70% of these private Sanger SNPs were observed below the 1% threshold and the remainder not observed within pyrosequences at all. This suggests the possibility that some private SS-SNPs correspond to sequencing errors rather than simply reflecting low sampling depth. This result was unexpected since each Sanger-derived viral genome sequence corresponds to the consensus of reads derived from a single viral template and thus should not include PCR errors.

An average of 43 PS-SNPs per subject across all the three genes (89% of all PS-SNPs detected) were observed at frequencies below their respective Sanger sequencing thresholds. However, as the majority of these SNPs were present at a frequency of <5%, diligence must be applied to minimize external sources of error such as using high-fidelity enzymes during amplification and incorporating parameters such as sequencing depth during data analysis to improve the accuracy of the observed polymorphisms. Pyrosequencing error patterns can skew minor variant distribution and frequencies [19, 26, 42]. However, following correction [19] the distribution of PS-SNPs adjacent to homopolymer and non-homopolymer regions showed no significant differences. Variant bias can also be introduced by PCR during viral template amplification [16, 49, 55]. Nonetheless, shared SNPs showed a high degree of correlation (average Spearman’s ρ of 0.87) between the two sequencing methods, suggesting that the impact of PCR bias in this study was minimal.

Sequencing depths of 1 or below are not adequate to resolve low frequency sequencing artifacts from genuine low frequency variants present within viral templates. An excess of reads compared to input viral templates will help fine-tune minor frequency SNP calls. Unfortunately, due to the uneven sequencing coverage observed with library sequencing [20, 25], more than 50% of the positions with PS-SNPs observed within the current dataset were located in regions with a sequencing depth < 2 (0.71% of all sequenced sites in the current dataset). Additionally, while the number of PS-SNPs were reduced in positions with higher sequencing depth, we did not observe significant changes in mean frequencies between PS-SNPs from positions with <5 or >5 depth in two of the three genome regions sequenced. An ideal comparison might be accomplished by analysis of pyrosequences from a genomic region with known viral templates and varying the sequencing depth through over-sequencing to quantitate the advantage of higher sequencing depth in resolving low frequency sequencing artifacts.

The concern over higher error rates, especially from the 454 pyrosequencing and Ion Torrent platforms, necessitates the application of a frequency threshold and additional filters in order to reduce or eliminate sequencing artifacts. The metric “sequencing depth” used here illustrates that increased read coverage with respect to number of amplifiable templates is associated with increased accuracy in the SNP frequencies at that position. As sequencing depth relies on read coverage and amplifiable templates, regions with poor read coverage or samples with large numbers of viral templates can decrease sequencing depth and subsequent confidence in the validity of observations of minor variants. Newer techniques such as PrimerID [49] and Duplex Sequencing [56] can lower the threshold of acceptable MPS depth.

The sequences generated through the two methods in this study differ with respect to primers and PCR procedures. The first study generated 9kb amplicons derived from single amplifiable viral RNA template molecules. This procedure is relatively inefficient compared to the PCR procedure used for the subsequent pyrosequencing study where multiplex amplification of three, 1.5Kb regions was performed. The study design required precious, earliest available specimens (only plasma specimens were available) from infected subjects enrolled in a large-scale vaccine trial. As a result there was no possibility of achieving the depth of population sampling through single template sequencing that could have made minor variants observed in pyrosequencing comparable to those observed through Sanger sequencing. Notwithstanding these differences, this study shows the adequacy of using Sanger sequencing for assessment of acute and very early HIV infection and serve as a guide regarding the design, utility and limitations of population sequencing variable template sources, and emphasize parameters for improving the interpretation of massively parallel sequencing data.

## Supporting Information

S1 Table PCR primers used in this study. *gag*, *gp120* and *nef* primers begin with Step, and then the letters G, E and N, respectively. Forward and reverse primers are indicated with an F or R, and first and second round primers are denoted with 1 and 2, respectively. A suffix of 0, 1 or 2 is used to denote whether that primer was the initial or alternate primer. Positions relative to the HXB2 reference sequence at the 5’ (R primers) or 3’ (F primers) ends are listed in the primer name.
(TIFF)

S2 Table Pyrosequencing-specific minor SNPs. Number and percent of pyrosequencing-specific minor SNPs (PS-SNPs) observed in all 32 subjects that fell below the Sanger sequencing detection threshold for that subject.
(TIFF)

S3 Table Pyrosequencing and Sanger sequence reads from *gag*. The median number of reads, mean amplifiable templates, mean sequencing depth, single-template derived Sanger sequences and plasma sample collection time is shown for each subject. The average gene length for *gag* is 1500 bases. The first available plasma sample was sequenced.
(TIFF)

S4 Table Pyrosequencing and Sanger sequence reads from *env-gp120*. The median number of reads, mean amplifiable templates, mean sequencing depth, single-template derived Sanger sequences and plasma sample collection time is shown for each subject. The average gene length for *gp120* is 1530 bases. The first available plasma sample was sequenced.
(TIFF)

S5 Table Pyrosequencing and Sanger sequence reads from *nef*. The median number of reads, mean amplifiable templates, mean sequencing depth, single-template derived Sanger sequences and plasma sample collection time is shown for each subject. The average gene length for *nef* is 610 bases. The first available plasma sample was sequenced.
(TIFF)

S6 Table Numbers of SNPs observed within the vaccine (N = 19 subjects) and placebo (N = 13 subjects) groups in the three regions sequenced. The fraction of SNPs observed within subjects in each of the categories was compared between vaccine and placebo groups. The categories compared include shared SNPs, SNPs observed specifically within pyrosequences (1–2%, 2–5%, 5–10%, 10–20%, 20–50%) and SNPs observed specifically within Sanger sequences (Private and Informative SNPs). The p values listed are based on Mann-Whitney comparison between vaccine and placebo subjects.
(TIFF)

S1 Fig SNP frequencies in subjects with multiple founders. The Y-axis indicates the proportion of nucleotide positions in *gag* (A; 1500nt), *gp120* (B; 1530nt), and *nef* (C; 615nt) that correspond to each SNP category, with a linear scale and a split at 0.25. The X-axis corresponds to each subject (ID 502-XXXX) [51]. The key indicates the types of SNPs observed.
(TIFF)

S2 Fig Number of sites with consensus base mismatches between Sanger and pyrosequences. Subjects with (A) single or (B) multiple founder viruses are shown. Subject IDs are indicated on the x-axis (502-XXXX) [51].
(TIFF)

S3 Fig Nucleotide frequencies at positions having consensus base mismatches between Sanger and pyrosequences. Positions with consensus mismatches in *gag* (A), *gp120* (B) and *nef* (C) and are shown. Subject IDs (502-XXXX)[51] are listed on the X-axis with blue lines separating each subject. The Y-axis shows the frequency of each discordant base. The key indicates the nucleotides observed.
(TIFF)

S4 Fig Fraction of minor SNPs detected in Sanger and pyrosequences in the six subjects with multiple founders. (A-C) Stacked bar graphs are shown for each subject with the key indicating the type of SNP and the frequency of each SNP in pyrosequences. The lower panels show the pyrosequencing depth (left y-axis), defined as number of reads mapped to a position divided by mean number of amplifiable viral templates. The number of Sanger sequences are shown on the right y-axis. X-axes list the subject publication ID (502-XXXX) [51].
(TIFF)

S5 Fig Proportion of pyrosequencing-specific minor SNPs found adjacent to homopolymer and non-homopolymer regions. The proportion of each subjects’ minor SNP variants found adjacent to a homopolymer (diamonds) or a nonhomopolymer (triangles) is shown on the Y-axis. Results from all 32 subjects and all three gene regions are shown. The P value derives from a Mann-Whitney comparison between the two groups.
(TIFF)

S6 Fig Reduction in pyrosequencing-specific minor SNPs upon exclusion of positions with a sequencing depth of <1. The number (Y-axis, logarithmic scale) of pyrosequencing-specific SNPs is plotted for all subjects (X-axis, 502-XXXX). All positions with minor SNPs (red) and minor SNP positions that have a sequencing depth greater than one (black) are shown for *gag* (A), *gp120* (B) and *nef* (C). Subjects replicating more than one founder variant correspond to the rightmost six columns in each plot.
(TIFF)

S7 Fig Frequency of Sanger-specific SNPs in a subject infected with multiple founders. Frequency of SNPs (left Y-axis) found in Sanger sequences, but absent from pyrosequences, in *gag*, *gp120* and *nef* (X-axis). The black line and right Y-axis shows the pyrosequencing depth at each SNP position.
(TIFF)

S8 Fig Frequency of private SS-SNPs as a function of SNP presence in the pyrosequencing dataset. The frequency of all private (observed in only one of a subject’s Sanger sequences) SNPs found in Sanger sequences across all 32 subjects in *gag* (A; n = 81), *gp120* (B; n = 116) and *nef* (C; n = 51) are plotted on the Y-axis, and binned according that SNP’s presence within pyrosequences. None of the Sanger sequence private SNPs were found in the pyrosequencing dataset above the 1% threshold.
(TIFF)

S9 Fig Shared SNPs in subjects with multiple founders. Error-corrected pyrosequences (Y-axis) and Sanger sequences (X-axis) among 103, 250, and 169 shared SNPs across *gag* (A), *gp120* (B) and *nef* (C) is shown for the six subjects with multiple founders. Major (+) and minor (o) variants are plotted. Spearman correlation coefficients are shown.
(TIFF)

S10 Fig Effect of sequencing depth on the absolute frequency difference of shared minor SNP variants in individuals with multiple founders. (A-C) Correlations between the absolute frequency difference and the pyrosequencing depth of the corresponding position in 103, 250, and 169 shared SNPs across *gag* (A), *gp120* (B) and *nef* (C) with Spearman correlation coefficients noted. (D-F) Correlations between the number of Sanger sequences and the absolute frequency difference for shared SNPs. Major (+) and minor (o) variants are plotted. Bars above each panel indicate the significance of correlations (* <0.05. ** <0.01, *** <0.001, **** <0.0001) using a Kruskal-Wallis test with Dunn correction for multiple comparisons.
(TIFF)

S11 Fig Reduction in shared minor SNPs upon exclusion of positions with a sequencing depth of <1. The number (Y-axis) of shared minor SNPs found in all subjects (X-axis). All positions with minor shared SNPs (orange) and only minor shared SNP positions that have a sequencing depth greater than one (turquoise) are shown for *gag* (A), *gp120* (B) and *nef* (C). Subjects infected with more than one founder are shown in the rightmost six columns in each plot.
(TIFF)

S12 Fig Frequencies of PS-SNPs in positions with varying sequencing depths. PS-SNPs observed in all single and multiple founder subjects across *gag* (A), *gp120* (B) and *nef* (C) are binned according to the estimated sequencing depth at that position. The correlation between frequencies of PS-SNPs and the sequencing depth of the position is estimated. Bars above each panel indicate the significance of correlations (* <0.05. ** <0.01, *** <0.001, **** <0.0001) using a Kruskal-Wallis test with Dunn correction for multiple comparisons. Means with SD (red bars) are shown.
(TIFF)
