# Supplementary figures and images for: Comparison of Major and Minor Viral SNPs Identified through Single Template Sequencing and Pyrosequencing in Acute HIV-1 Infection
Source: PLoS One. 2015 Aug 28;10(8):e0135903. doi: 10.1371/journal.pone.0135903 (PMC4552882; doi:10.1371/journal.pone.0135903)

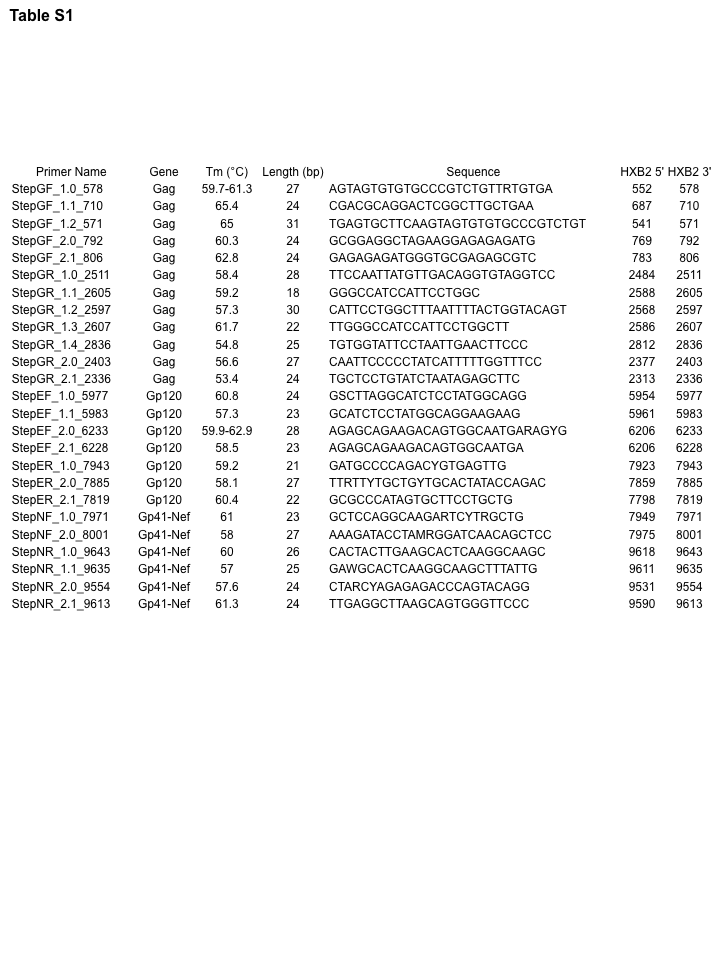

Supplement: S1 Table — gag, gp120 and nef primers begin with Step, and then the letters G, E and N, respectively. Forward and reverse primers are indicated with an F or R, and first and second round primers are denoted with 1 and 2, respectively. A suffix of 0, 1 or 2 is used to denote whether that primer was the initial or alternate primer. Positions relative to the HXB2 reference sequence at the 5’ (R primers) or 3’ (F primers) ends are listed in the primer name. (TIFF) [file pone.0135903.s001.tiff]

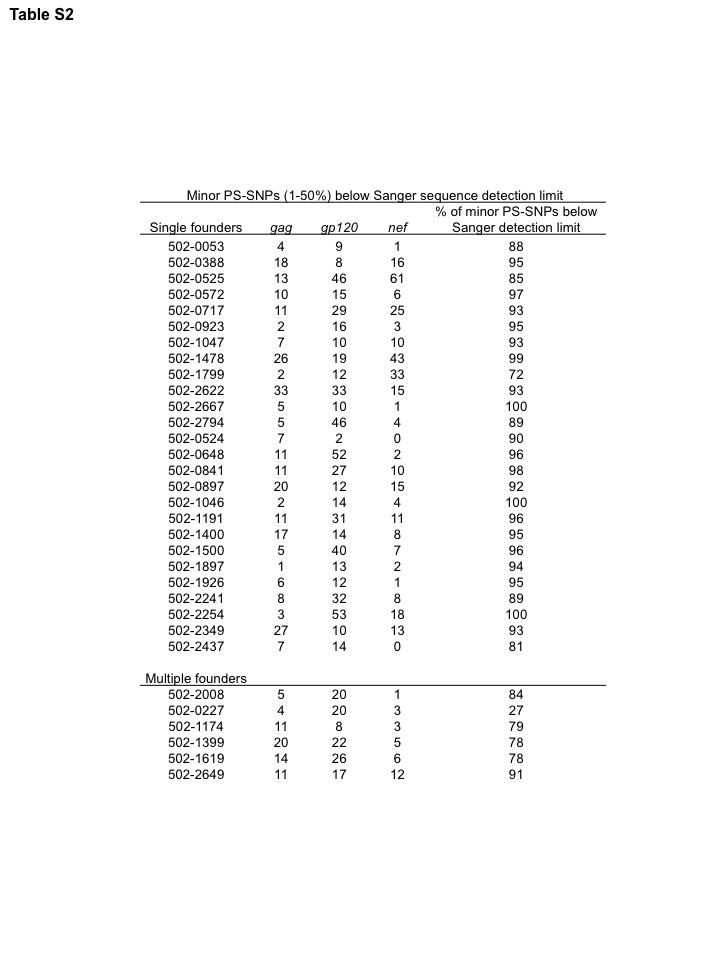

Supplement: S2 Table — Number and percent of pyrosequencing-specific minor SNPs (PS-SNPs) observed in all 32 subjects that fell below the Sanger sequencing detection threshold for that subject. (TIFF) [file pone.0135903.s002.tiff]

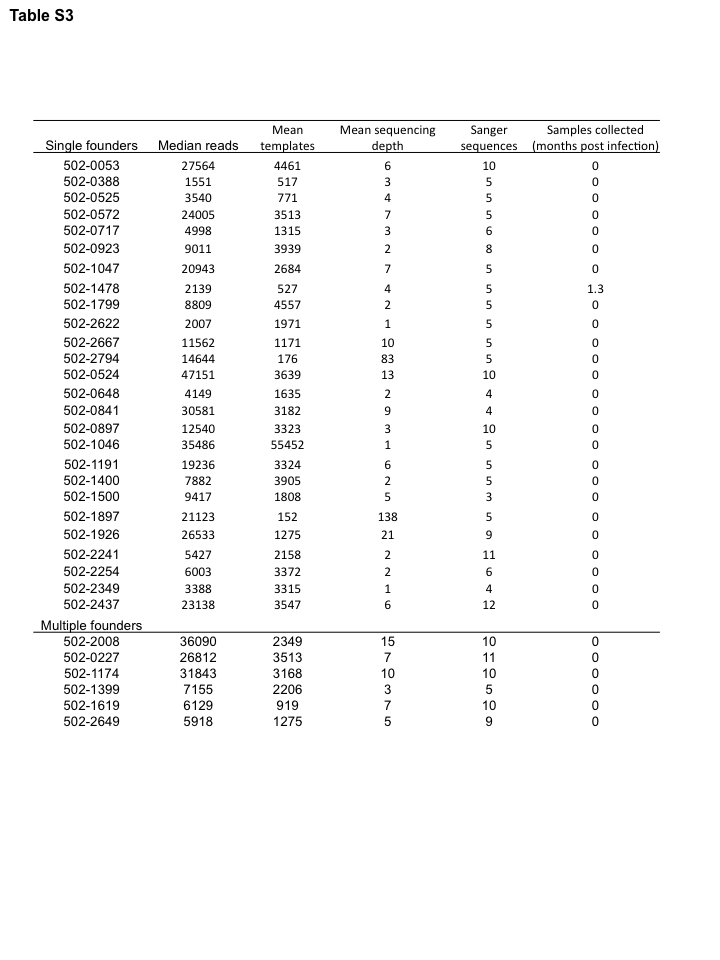

Supplement: S3 Table — The median number of reads, mean amplifiable templates, mean sequencing depth, single-template derived Sanger sequences and plasma sample collection time is shown for each subject. The average gene length for gag is 1500 bases. The first available plasma sample was sequenced. (TIFF) [file pone.0135903.s003.tiff]

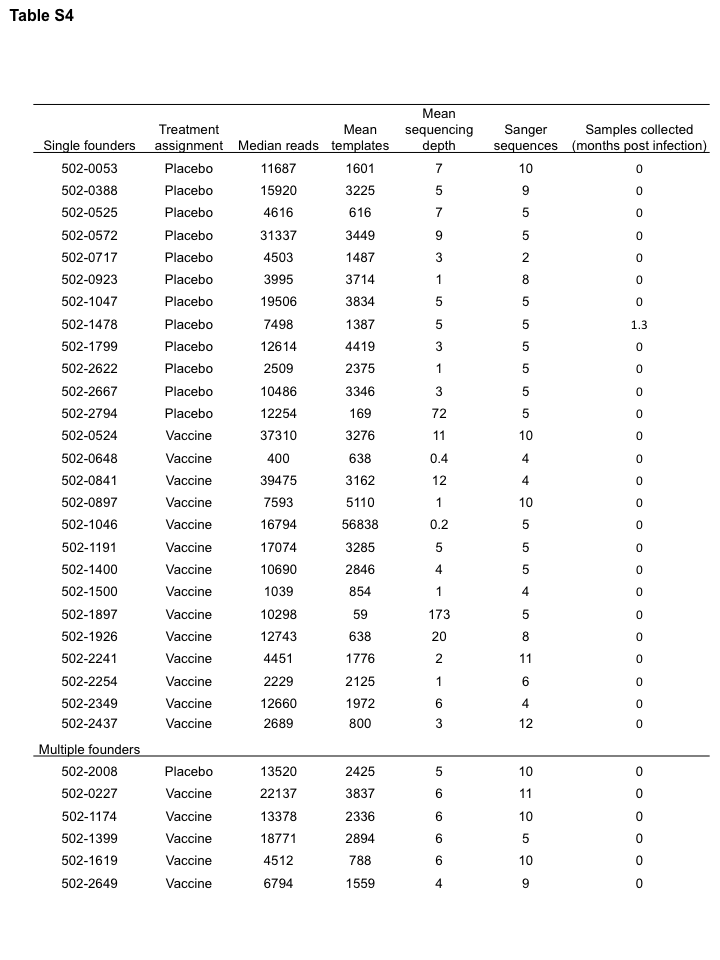

Supplement: S4 Table — The median number of reads, mean amplifiable templates, mean sequencing depth, single-template derived Sanger sequences and plasma sample collection time is shown for each subject. The average gene length for gp120 is 1530 bases. The first available plasma sample was sequenced. (TIFF) [file pone.0135903.s004.tiff]

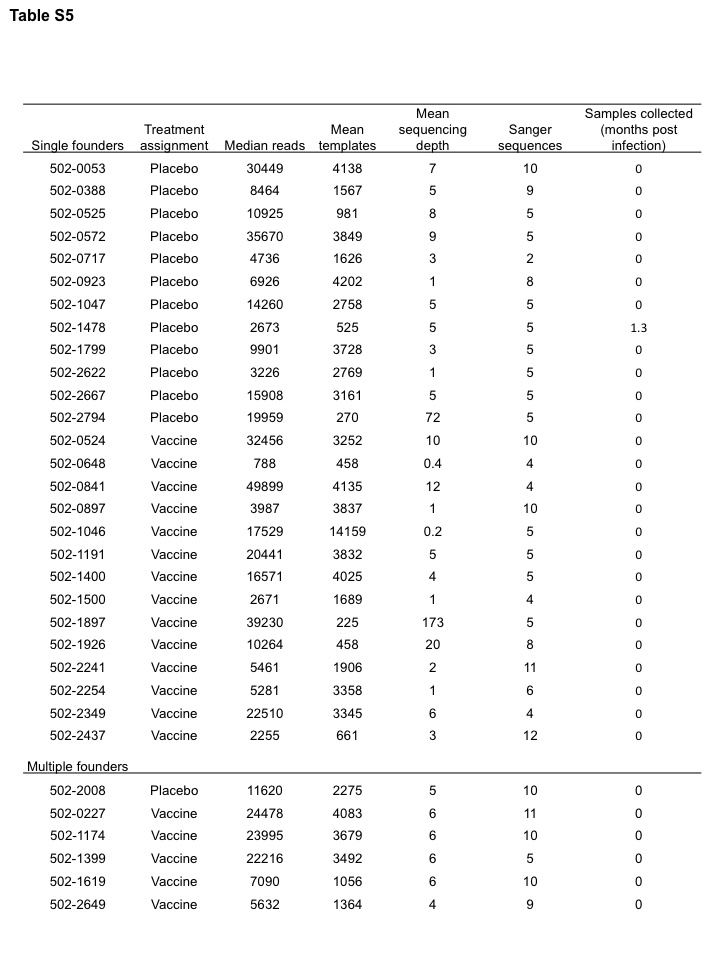

Supplement: S5 Table — The median number of reads, mean amplifiable templates, mean sequencing depth, single-template derived Sanger sequences and plasma sample collection time is shown for each subject. The average gene length for nef is 610 bases. The first available plasma sample was sequenced. (TIFF) [file pone.0135903.s005.tiff]

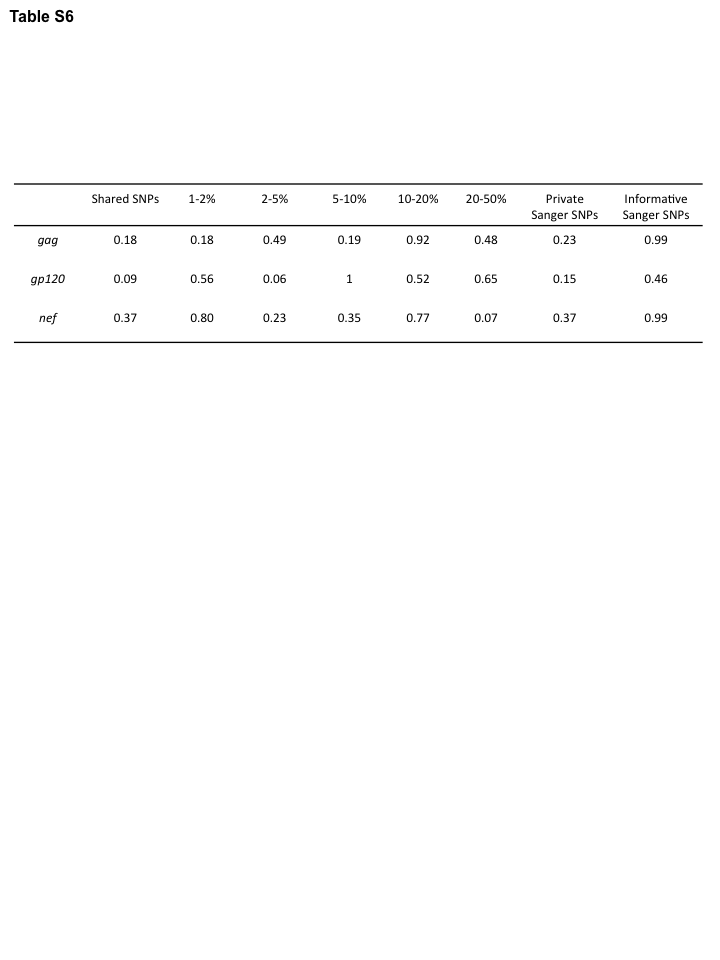

Supplement: S6 Table — The fraction of SNPs observed within subjects in each of the categories was compared between vaccine and placebo groups. The categories compared include shared SNPs, SNPs observed specifically within pyrosequences (1–2%, 2–5%, 5–10%, 10–20%, 20–50%) and SNPs observed specifically within Sanger sequences (Private and Informative SNPs). The p values listed are based on Mann-Whitney comparison between vaccine and placebo subjects. (TIFF) [file pone.0135903.s006.tiff]

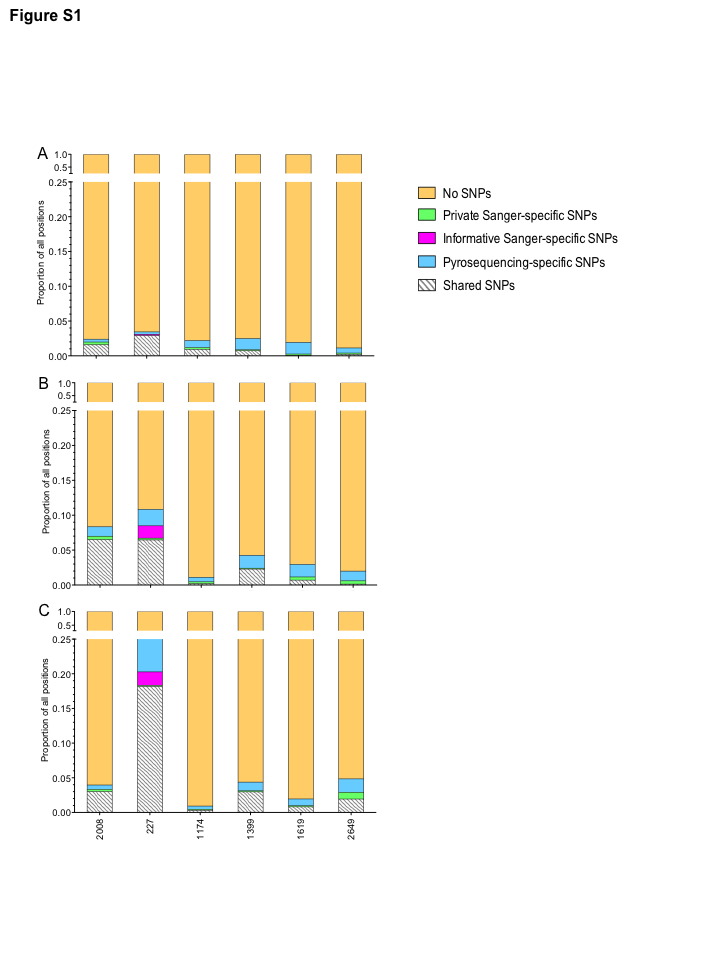

Supplement: S1 Fig — The Y-axis indicates the proportion of nucleotide positions in gag (A; 1500nt), gp120 (B; 1530nt), and nef (C; 615nt) that correspond to each SNP category, with a linear scale and a split at 0.25. The X-axis corresponds to each subject (ID 502-XXXX) [51]. The key indicates the types of SNPs observed. (TIFF) [file pone.0135903.s007.tiff]

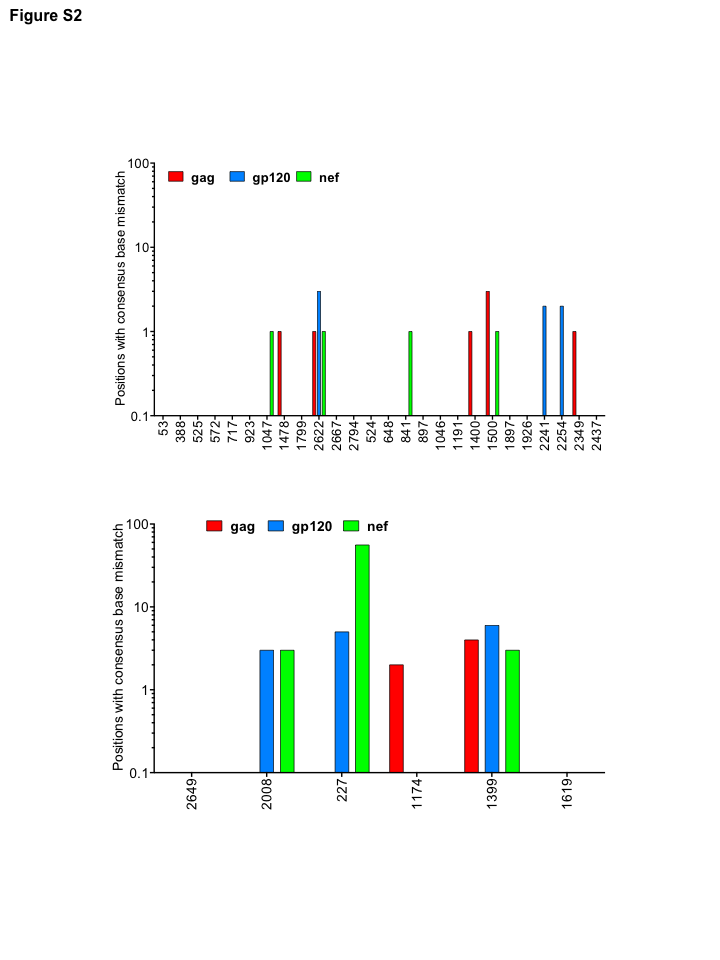

Supplement: S2 Fig — Subjects with (A) single or (B) multiple founder viruses are shown. Subject IDs are indicated on the x-axis (502-XXXX) [51]. (TIFF) [file pone.0135903.s008.tiff]

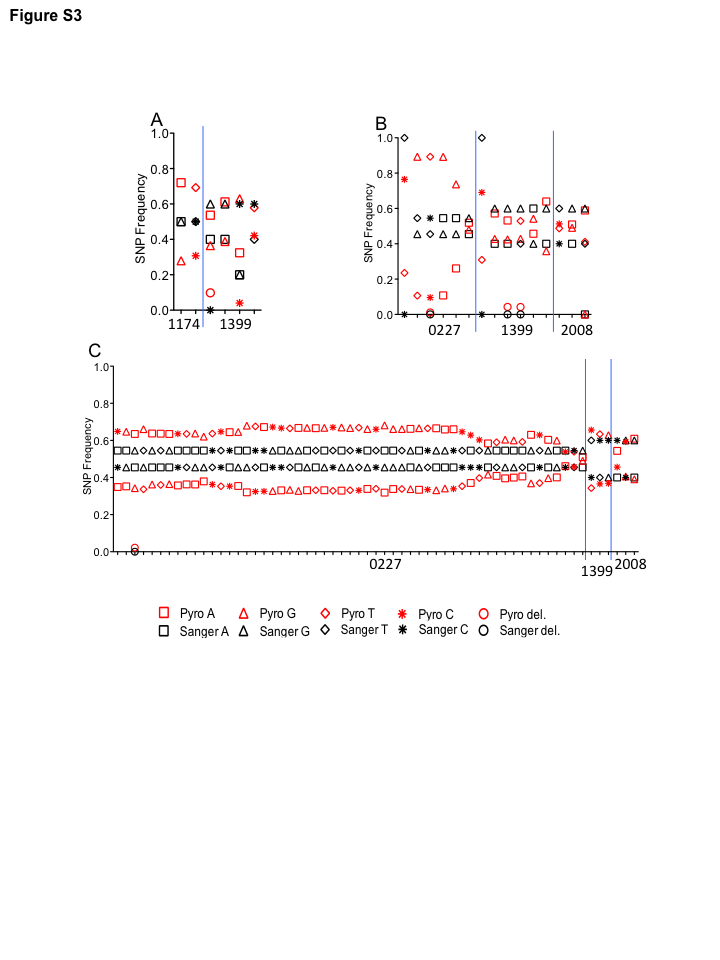

Supplement: S3 Fig — Positions with consensus mismatches in gag (A), gp120 (B) and nef (C) and are shown. Subject IDs (502-XXXX)[51] are listed on the X-axis with blue lines separating each subject. The Y-axis shows the frequency of each discordant base. The key indicates the nucleotides observed. (TIFF) [file pone.0135903.s009.tiff]

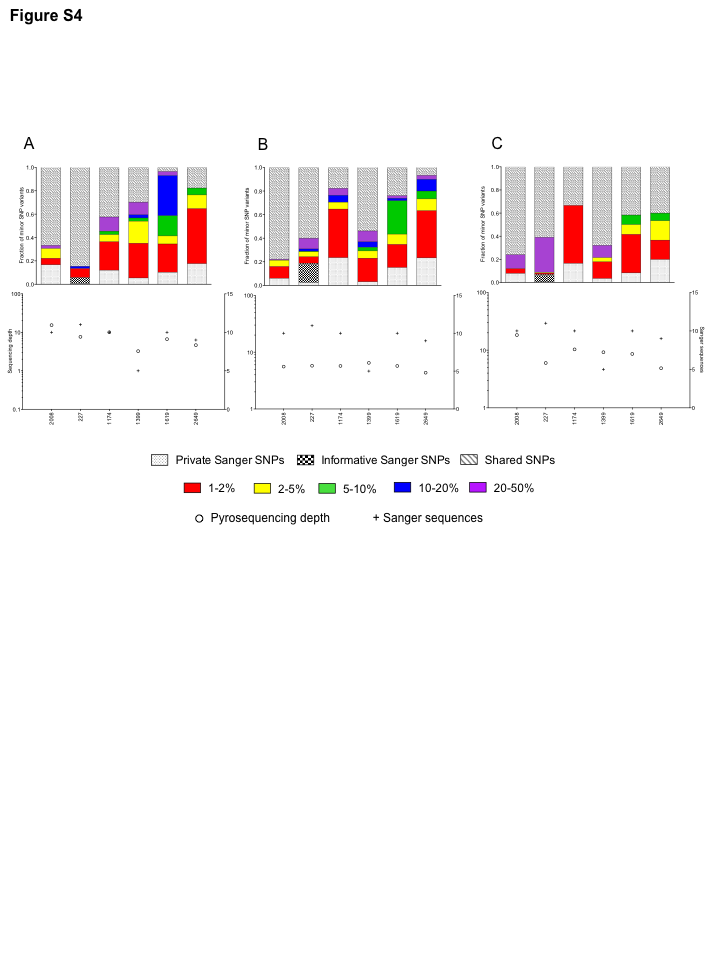

Supplement: S4 Fig — (A-C) Stacked bar graphs are shown for each subject with the key indicating the type of SNP and the frequency of each SNP in pyrosequences. The lower panels show the pyrosequencing depth (left y-axis), defined as number of reads mapped to a position divided by mean number of amplifiable viral templates. The number of Sanger sequences are shown on the right y-axis. X-axes list the subject publication ID (502-XXXX) [51]. (TIFF) [file pone.0135903.s010.tiff]

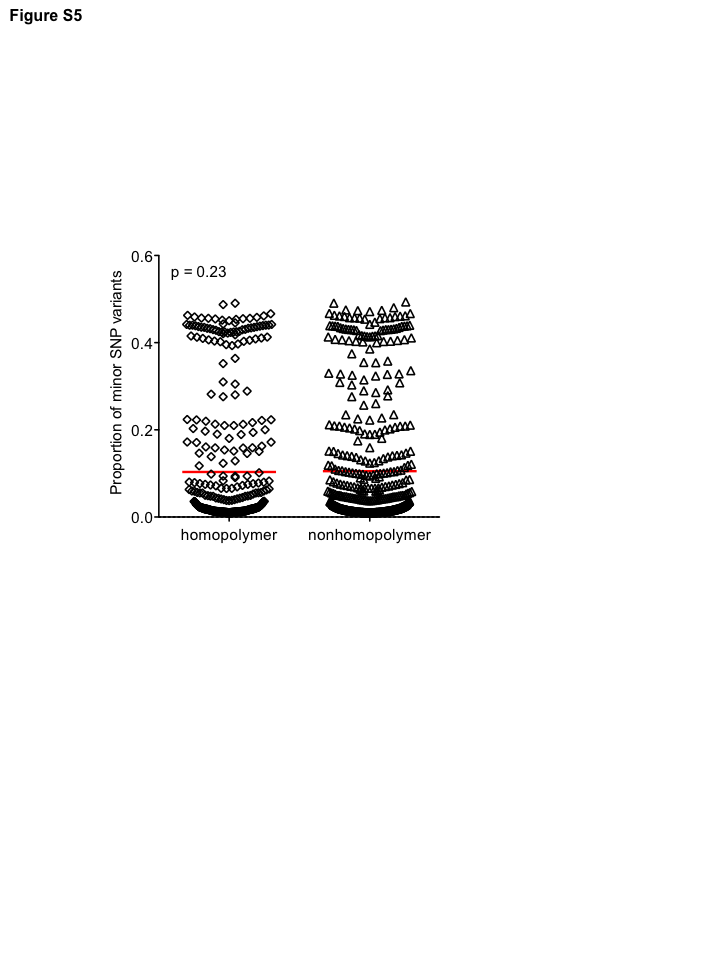

Supplement: S5 Fig — The proportion of each subjects’ minor SNP variants found adjacent to a homopolymer (diamonds) or a nonhomopolymer (triangles) is shown on the Y-axis. Results from all 32 subjects and all three gene regions are shown. The P value derives from a Mann-Whitney comparison between the two groups. (TIFF) [file pone.0135903.s011.tiff]

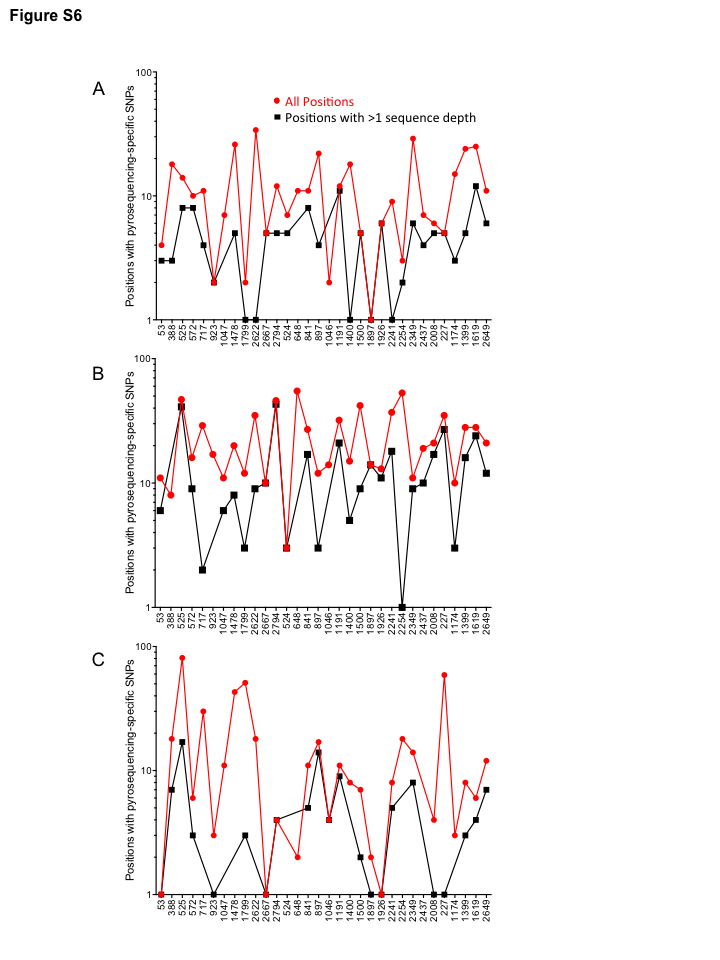

Supplement: S6 Fig — The number (Y-axis, logarithmic scale) of pyrosequencing-specific SNPs is plotted for all subjects (X-axis, 502-XXXX). All positions with minor SNPs (red) and minor SNP positions that have a sequencing depth greater than one (black) are shown for gag (A), gp120 (B) and nef (C). Subjects replicating more than one founder variant correspond to the rightmost six columns in each plot. (TIFF) [file pone.0135903.s012.tiff]

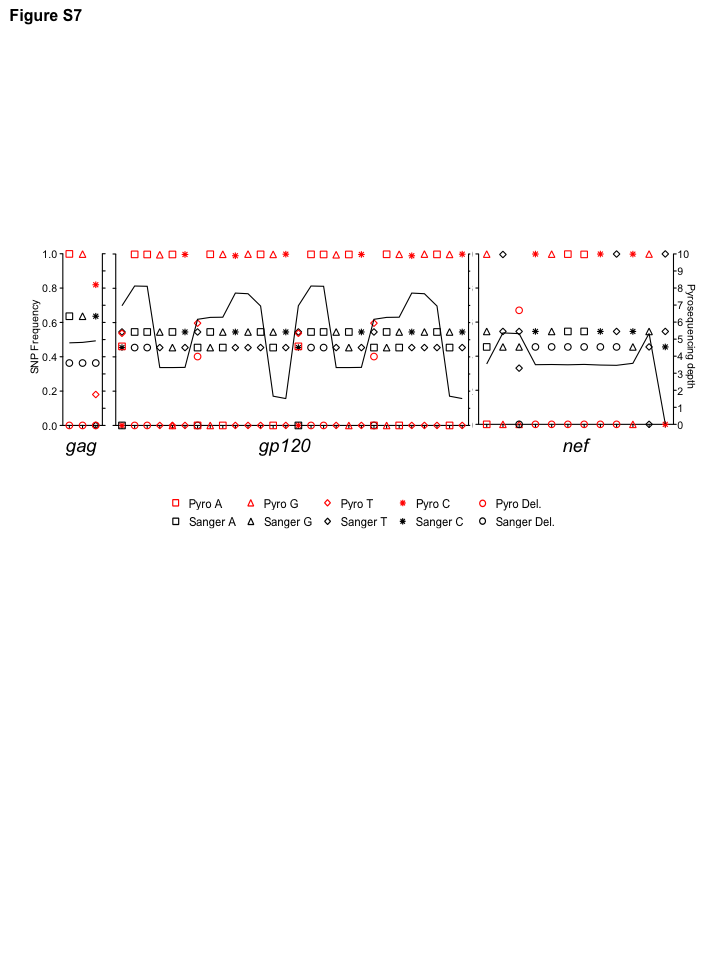

Supplement: S7 Fig — Frequency of SNPs (left Y-axis) found in Sanger sequences, but absent from pyrosequences, in gag, gp120 and nef (X-axis). The black line and right Y-axis shows the pyrosequencing depth at each SNP position. (TIFF) [file pone.0135903.s013.tiff]

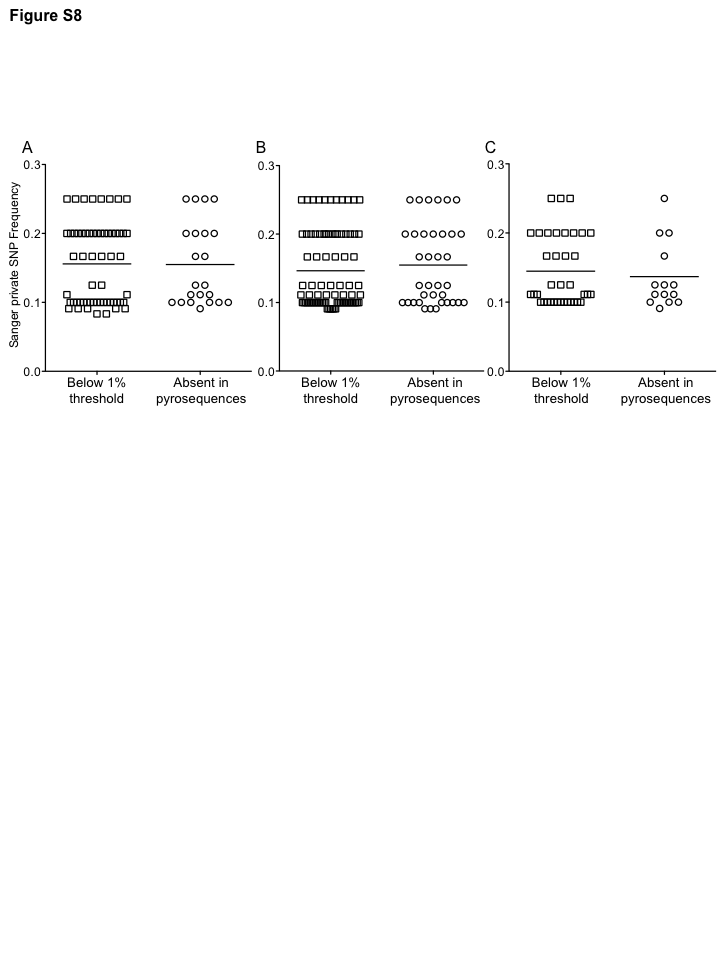

Supplement: S8 Fig — The frequency of all private (observed in only one of a subject’s Sanger sequences) SNPs found in Sanger sequences across all 32 subjects in gag (A; n = 81), gp120 (B; n = 116) and nef (C; n = 51) are plotted on the Y-axis, and binned according that SNP’s presence within pyrosequences. None of the Sanger sequence private SNPs were found in the pyrosequencing dataset above the 1% threshold. (TIFF) [file pone.0135903.s014.tiff]

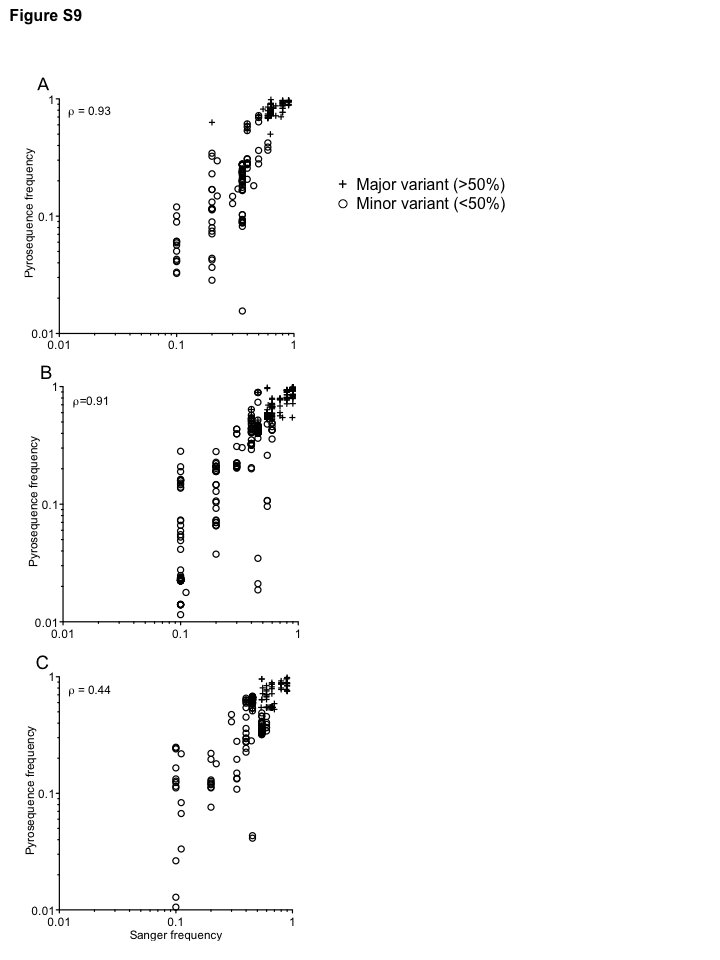

Supplement: S9 Fig — Error-corrected pyrosequences (Y-axis) and Sanger sequences (X-axis) among 103, 250, and 169 shared SNPs across gag (A), gp120 (B) and nef (C) is shown for the six subjects with multiple founders. Major (+) and minor (o) variants are plotted. Spearman correlation coefficients are shown. (TIFF) [file pone.0135903.s015.tiff]

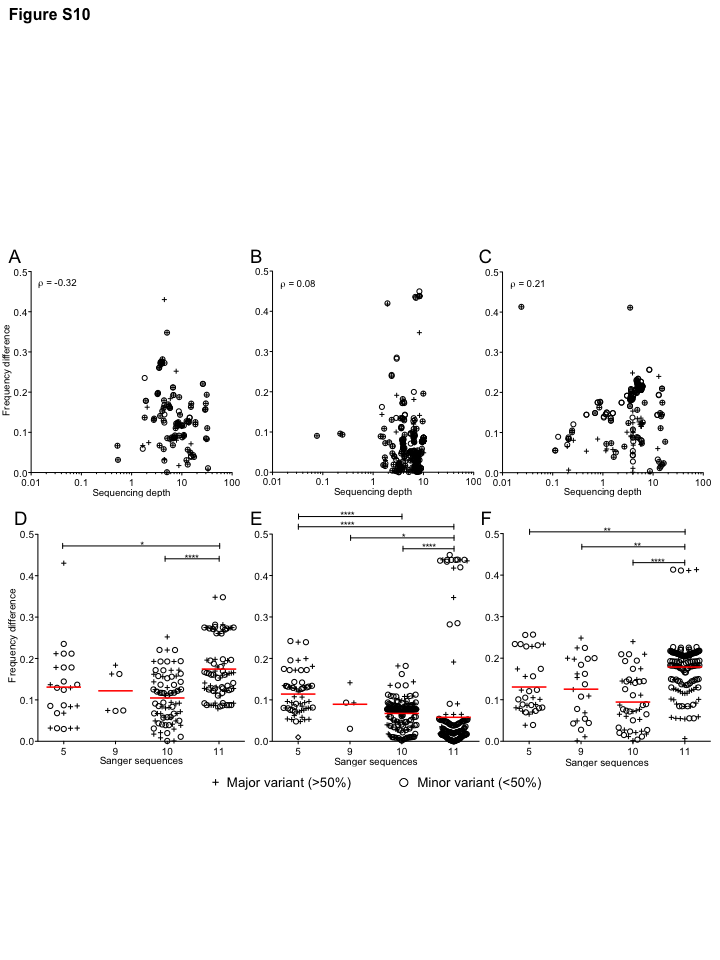

Supplement: S10 Fig — (A-C) Correlations between the absolute frequency difference and the pyrosequencing depth of the corresponding position in 103, 250, and 169 shared SNPs across gag (A), gp120 (B) and nef (C) with Spearman correlation coefficients noted. (D-F) Correlations between the number of Sanger sequences and the absolute frequency difference for shared SNPs. Major (+) and minor (o) variants are plotted. Bars above each panel indicate the significance of correlations (* <0.05. ** <0.01, *** <0.001, **** <0.0001) using a Kruskal-Wallis test with Dunn correction for multiple comparisons. (TIFF) [file pone.0135903.s016.tiff]

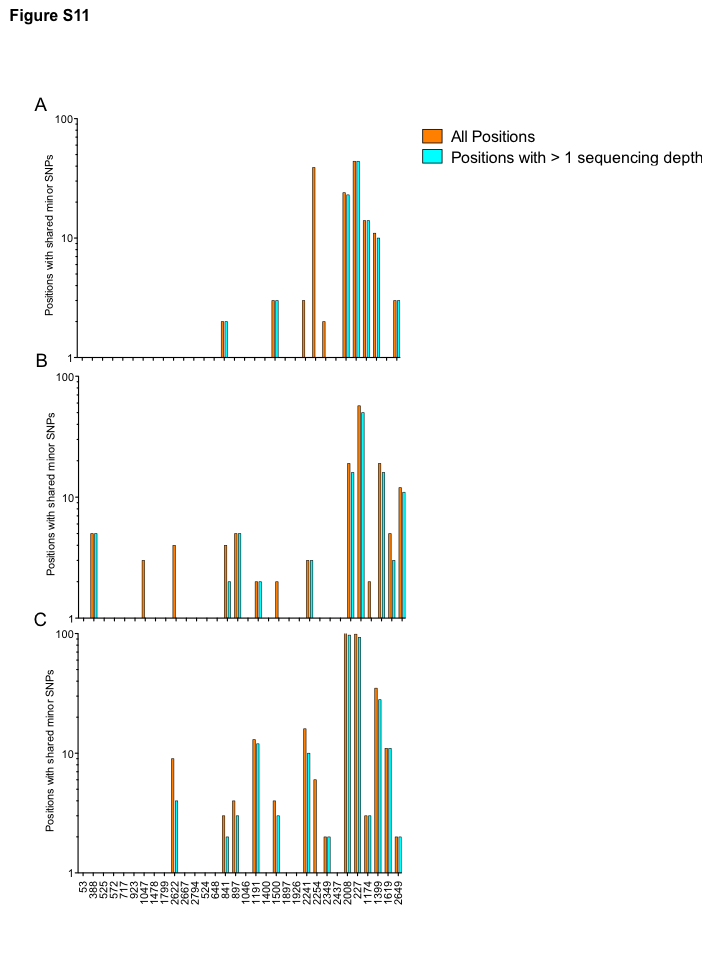

Supplement: S11 Fig — The number (Y-axis) of shared minor SNPs found in all subjects (X-axis). All positions with minor shared SNPs (orange) and only minor shared SNP positions that have a sequencing depth greater than one (turquoise) are shown for gag (A), gp120 (B) and nef (C). Subjects infected with more than one founder are shown in the rightmost six columns in each plot. (TIFF) [file pone.0135903.s017.tiff]

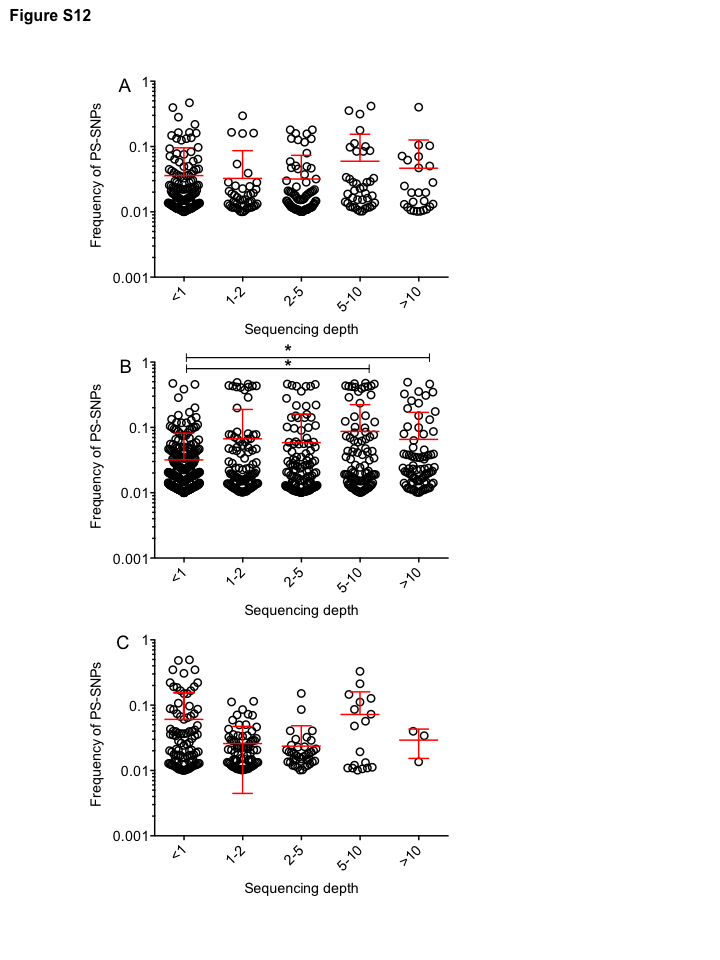

Supplement: S12 Fig — PS-SNPs observed in all single and multiple founder subjects across gag (A), gp120 (B) and nef (C) are binned according to the estimated sequencing depth at that position. The correlation between frequencies of PS-SNPs and the sequencing depth of the position is estimated. Bars above each panel indicate the significance of correlations (* <0.05. ** <0.01, *** <0.001, **** <0.0001) using a Kruskal-Wallis test with Dunn correction for multiple comparisons. Means with SD (red bars) are shown. (TIFF) [file pone.0135903.s018.tiff]
